# Wiederaufnahmen auf die Intensivstation: Seltenes Phänomen oder relevant für die klinische Praxis?

**DOI:** 10.1007/s00063-026-01441-6

**Published:** 2026-04-20

**Authors:** Denise Schindele, Irmela Gnass, Marc Moritz Berger

**Affiliations:** 1 Institut für Pflegewissenschaft und -praxis, Zentrum für Public Health und Versorgungsforschung, Paracelsus Medizinische Privatuniversität, Strubergasse 21, 5020 Salzburg, Österreich; 2 Regionale Kliniken Holding & Services GmbH, RKH-Akademie, Ludwigsburg, Deutschland; 3 Klinik für Anästhesiologie, Intensivmedizin, Notfallmedizin und Schmerztherapie, RKH Klinikum Ludwigsburg, Ludwigsburg, Deutschland

**Keywords:** Behandlungsqualität, Gebrechlichkeit, Sterblichkeit, Verweildauer, Patientenentlassung, Quality of health care, Frailty, Mortality, Length of stay, Patient discharge

## Abstract

**Hintergrund:**

Ungeplante Wiederaufnahmen auf die Intensivstation (ITS) sind selten, jedoch klinisch hochrelevant. Trotz ihrer klinischen Bedeutung sind die zugrunde liegenden Risikofaktoren und Mechanismen von Wiederaufnahmen bislang nur unzureichend charakterisiert.

**Ziel:**

Ziel dieses Übersichtsbeitrags ist es, den aktuellen Kenntnisstand zu ungeplanten ITS-Wiederaufnahmen darzustellen, assoziierte Risikofaktoren und Ursachen zu identifizieren sowie deren Bedeutung für Mortalität und Ressourcenverbrauch einzuordnen.

**Material und Methoden:**

Es erfolgte eine systematische Literaturrecherche in PubMed und CINAHL. Eingeschlossen wurden englisch- und deutschsprachige Publikationen der letzten fünf Jahre zur Abbildung des aktuellen Evidenzstands. Die eingeschlossenen Publikationen untersuchten ungeplante ITS-Wiederaufnahmen erwachsener Patienten während desselben Krankenhausaufenthalts. Neun retrospektive Kohortenstudien wurden identifiziert und aufgrund methodischer Heterogenität narrativ ausgewertet.

**Ergebnisse:**

Die berichteten Wiederaufnahmeraten lagen zwischen 3,6 und 9,1 %. Die Studien untersuchten überwiegend Patientenkollektive aus universitären Zentren und Akutkrankenhäusern der Maximalversorgung mit interdisziplinären oder spezialisierten Intensivstationen. Wiederaufnahmen waren konsistent mit einer erhöhten Krankenhaus- und Intensivstationsmortalität sowie verlängerten Krankenhaus- und Intensivaufenthalten assoziiert. Relevante Risikofaktoren umfassten höheres Lebensalter, funktionelle Einschränkungen, kognitive Störungen, persistierende Organfunktionsstörungen sowie prolongierte Organunterstützung. Neben patienten- und krankheitsbezogenen Faktoren deuten die Daten zudem auf einen möglichen Einfluss struktureller und organisatorischer Rahmenbedingungen der Intensiventlassung hin. Häufigste Ursachen der Wiederaufnahme waren respiratorische Komplikationen, gefolgt von hämodynamischer Instabilität sowie infektiösen und neurologischen Verschlechterungen.

**Schlussfolgerung:**

Ungeplante ITS-Wiederaufnahmen betreffen je nach Setting zwischen 3,6 und 9,1 % der intensivmedizinisch behandelten Patienten und sind klinisch relevant. Sie reflektieren ein komplexes Zusammenspiel aus Patientenvulnerabilität, Krankheitsschwere und Versorgungsfaktoren und sind mit einer erhöhten Mortalität verbunden.

## Abstract (en)

**Background:**

Unplanned intensive care unit (ICU) readmissions are infrequent but clinically highly relevant. Despite their clinical importance, the underlying risk factors and mechanisms leading to unplanned readmissions remain insufficiently characterized.

**Aim:**

This narrative review aims to summarize the current evidence on unplanned ICU readmissions, identify associated risk factors and causes, and contextualize their relevance for mortality and resource utilization.

**Materials and methods:**

A systematic literature search was conducted in PubMed and CINAHL. Publications in English or German published within the past 5 years were included to reflect the current state of evidence. Eligible studies investigated unplanned ICU readmissions of adult patients during the same hospital admission. Nine retrospective cohort studies were identified and narratively synthesized due to methodological heterogeneity.

**Results:**

Reported ICU readmission rates ranged from 3.6% to 9.1%. The included studies predominantly investigated patient populations treated at university-affiliated centers and acute care hospitals with interdisciplinary or specialized ICUs. ICU readmissions were consistently associated with increased hospital and ICU mortality, as well as prolonged hospital and ICU length of stay. Relevant risk factors included advanced age, functional impairment, cognitive disturbances, persistent organ dysfunction, and prolonged organ support. The most common causes of readmission were respiratory complications, followed by hemodynamic instability and infectious or neurological deterioration.

**Conclusions:**

Unplanned ICU readmissions are infrequent but clinically highly relevant. They reflect a complex interaction of patient vulnerability, disease severity, and care-related factors, and are associated with increased mortality.

Ungeplante Wiederaufnahmen auf die Intensivstation (ITS) stellen ein klinisch bedeutsames Ereignis dar. Im Kontext ungeplanter Wiederaufnahmen stellt sich die Frage nach den zugrunde liegenden Ursachen und Risikofaktoren. Dabei rücken neben patienten- und krankheitsbezogenen Aspekten insbesondere die strukturellen Rahmenbedingungen der ITS-Entlassung und postintensivmedizinischen Versorgung in den Fokus der klinischen Forschung.

Ungeplante Wiederaufnahmen auf die ITS stellen ein relevantes Ereignis für kritisch kranke Patienten dar. Patienten mit einer ungeplanten Wiederaufnahme auf die ITS weisen eine erhöhte Morbidität, eine verlängerte Krankenhausverweildauer sowie eine erhöhte Krankenhaus- und ITS-Mortalität auf [4, 5, 8].

Strukturelle Faktoren beeinflussen die Vulnerabilität der Patienten nach einem Intensivaufenthalt

Die Entscheidung zur Entlassung eines Patienten von der ITS erfolgt häufig unter komplexen klinischen und organisatorischen Rahmenbedingungen und ist nicht allein durch den aktuellen Gesundheitszustand bestimmt. Neben der individuellen Krankheitsschwere können strukturelle Faktoren wie Bettenverfügbarkeit oder zeitlicher Entlassungsdruck Einfluss auf den Entlassungszeitpunkt nehmen und die Vulnerabilität der Patienten im postintensivmedizinischen Verlauf erhöhen [8, 16]. Bei eingeschränkten intensivmedizinischen Kapazitäten rückt die sichere Gestaltung des Übergangs von der ITS auf nachgelagerte Stationen zunehmend in den Fokus. Unabhängig von der absoluten Bettenkapazität können hohe Auslastung, personelle Ressourcenbegrenzungen oder organisatorische Rahmenbedingungen den Entscheidungsdruck im Rahmen von Intensiventlassungen erhöhen [6].

Obwohl Wiederaufnahmen auf die ITS seit Langem Gegenstand wissenschaftlicher Untersuchungen sind, bestehen weiterhin Unklarheiten hinsichtlich der relativen Bedeutung patientenbezogener und organisatorischer Risikofaktoren sowie in Bezug auf deren Zusammenhang mit dem klinischen Outcome. Zudem erschweren Unterschiede in Studiendesign, Patientenpopulationen und Definitionen der Wiederaufnahme eine einheitliche Interpretation der verfügbaren Daten [4, 5, 8, 16].

Vor diesem Hintergrund zielt der vorliegende Übersichtsbeitrag darauf ab, den aktuellen Kenntnisstand zu ungeplanten Wiederaufnahmen auf die ITS darzustellen, relevante Risikofaktoren aufzuzeigen und deren Bedeutung für Morbidität, Mortalität und Ressourcenverbrauch kritisch einzuordnen.

## Methode

Von November bis Dezember 2025 erfolgte eine systematische Literaturrecherche in den Datenbanken PubMed und CINAHL zur Identifikation von Studien zu ungeplanten Wiederaufnahmen auf die ITS während desselben Krankenhausaufenthalts bei erwachsenen Patienten. Für die Entwicklung der Suchstrings wurden MeSH-Terms (patient readmission, intensive care units, critical care outcomes, risk factors, mortality, length of stay) sowie CINAHL Headings verwendet und iterativ angepasst. Die Schlüsselbegriffe wurden mithilfe boolescher Operatoren kombiniert.

Einschlusskriterien:

- Patienten ≥ 18 Jahre
- Deutsch- oder englischsprachige Publikationen
- Veröffentlichungen nach Peer-Review-Prozess
- Empirische Studien
- Untersuchungen im Kontext der ITS
- Fokus auf Wiederaufnahmen auf die ITS
- Publikationen der letzten 5 Jahre zur Darstellung der Aktualität

Ausschlusskriterien:

- Patienten < 18 Jahren
- Graue Literatur (nicht verlagsgebundene Veröffentlichungen)
- Studien ohne ITS-Kontext
- Wiederaufnahmen ins Krankenhaus
- Erste Aufnahme auf die ITS

**Abb. 1:**
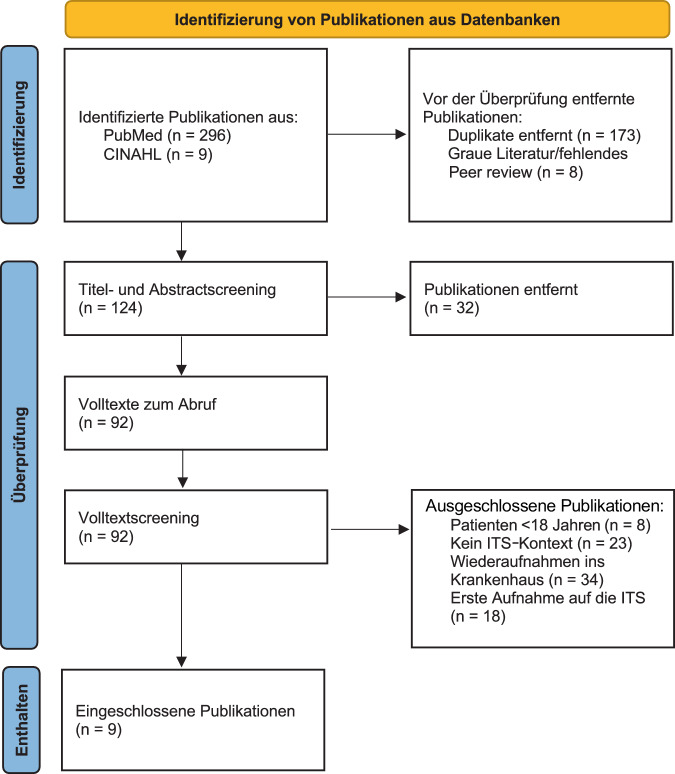
PRISMA-Flussdiagramm, Ergebnisdarstellung des Literaturscreenings nach [12]. *ITS* Intensivstation, *PRISMA* Preferred Reporting Items for Systematic Reviews and Meta-Analyses

Die Studienselektion erfolgte in einem zweistufigen Screeningprozess (Titel/Abstract, Volltext) anhand der definierten Ein- und Ausschlusskriterien. Die Datenextraktion erfolgte unter Nutzung eines standardisierten Datenextraktions-Sheets mit Fokus auf beschriebenen Risikofaktoren für eine ITS-Wiederaufnahme sowie auf dem Zusammenhang zwischen Wiederaufnahme und patientenrelevanten Outcomes. Insgesamt wurden 9 Studien in die Analyse eingeschlossen (Abb. 1). Aufgrund der Heterogenität der Studien erfolgte eine narrative Zusammenfassung der Ergebnisse.

## Ergebnisse

### Studiencharakteristika und Wiederaufnahmeraten

Die in den eingeschlossenen Studien berichteten Raten für Wiederaufnahmen auf die ITS während desselben Krankenhausaufenthalts zeigten eine breite Spanne abhängig von Patientenpopulation, klinischem Setting und Studiendesign [1–3, 10, 11, 13–15, 17]. Insgesamt wurden 9 retrospektive Kohortenstudien eingeschlossen. Die Studien wurden an universitären Zentren und Häusern der Maximalversorgung durchgeführt. Die Stichprobengrößen lagen zwischen 739 und 185.241 Patienten. Die berichteten Wiederaufnahmeraten lagen zwischen 3,6 und 9,1 % (isolierter Endpunkt: ungeplante Wiederaufnahme während desselben Aufenthalts; [1–3, 10, 11, 14, 17]). Die Definition der Wiederaufnahme war zwischen den Studien nicht einheitlich. Während die Mehrheit der Arbeiten eine Wiederaufnahme während desselben Krankenhausaufenthalts untersuchte [1, 10, 11, 14, 17], differenzierte eine Studie zusätzlich hinsichtlich Zeitintervallen nach Intensiventlassung (≤ 24 h, ≤ 48 h oder ≤ 72 h; [2]). Zwei Studien verwendeten hingegen einen kombinierten Endpunkt aus Wiederaufnahme auf die ITS oder Tod auf der Normalstation nach Intensiventlassung und berichteten entsprechend Ereignisraten von 4,7 % bzw. 5,3 %, die nicht als isolierte Wiederaufnahmeraten interpretierbar sind [13, 15].

Diese methodischen Unterschiede limitieren die direkte Vergleichbarkeit der berichteten Wiederaufnahmeraten, verdeutlichen jedoch zugleich die Heterogenität der untersuchten klinischen Konstellationen. Eine zusammenfassende Übersicht der Studiencharakteristika, Wiederaufnahmedefinitionen und -raten sowie weiterer extrahierter Variablen (Risikofaktoren, Ursachen der Wiederaufnahme und Outcomeparameter) ist in Tab. 1 dargestellt.

**Tab. 1:** Ergebnisse Datenextraktion

Autor (Jahr)	Land	Studiendesign	Stichprobe	Population/Setting	Definition Wiederaufnahme	Rate Wiederaufnahme (%)	Mit Wiederaufnahme assoziierte Risikofaktoren^2^	Ursachen der ITS-Wiederaufnahme (berichtete Indikationen)	Mit Wiederaufnahme signifikant assoziierte Outcomes
Agoubi et al. (2024; [1])	USA	Retrospektive Kohortenstudie, Single-Center-Studie	6691	≥ 65 Jahre, Trauma-ITS	Wiederaufnahme während desselben Krankenhausaufenthalts	9,1 % (*n* = 339)	Delir; Aspiration	Respiratorische Komplikationen; Aspiration	Erhöhte Krankenhausmortalität
Amagai et al. (2025; [2])	USA	Retrospektive Kohortenstudie, Multicenterstudie	185.241	≥ 18 Jahre, interdisziplinäre ITS	Ungeplante Wiederaufnahme während desselben Krankenhausaufenthalts; ≤ 24 h, ≤ 48 h, ≤ 72 h	8,6 % (*n* = 15.846)^1^	Höheres Alter; längerer initialer ITS-Aufenthalt	Erhöhter Bedarf an respiratorischer und hämodynamischer Unterstützung bei Wiederaufnahme	Erhöhte Krankenhausmortalität; erhöhter Bedarf an respiratorischer und hämodynamischer Unterstützung
Brosseau et al. (2024; [3])	Kanada	Retrospektive Kohortenstudie, Multicenterstudie	8017	≥18 Jahre, interdisziplinäre ITS	Wiederaufnahme > 24 h nach ITS-Entlassung	3,6 % (*n* = 291)	Unfähigkeit zu stehen bei ITS-Entlassung; höheres Alter; Multimorbidität; längerer initialer ITS-Aufenthalt	Nicht berichtet	Erhöhte Krankenhausmortalität; längere ITS-Verweildauer
Moore et al. (2025; [10])	USA	Retrospektive Kohortenstudie, Single-Center-Studie	3632	≥ 18 Jahre, Trauma-ITS	Wiederaufnahme während desselben Krankenhausaufenthalts	7,7 % (*n* = 278)	Höheres Alter; invasive Beatmung; längere Beatmungsdauer	Respiratorische; kardiovaskuläre; neurologische Ursachen	Längere Krankenhausverweildauer
Padkins et al. (2022; [11])	USA	Retrospektive Kohortenstudie, Single-Center-Studie	9434	≥ 18 Jahre, kardiologische ITS	Wiederaufnahme nach Verlegung auf Normalstation während desselben Aufenthalts	1,5 % (*n* = 138)	Respiratorisches Versagen; schweres akutes Nierenversagen; höhere Komorbiditätslast	Respiratorisches Versagen; prozedur-/operationsassoziierte Komplikationen	Erhöhte Krankenhausmortalität; erhöhte 1‑Jahres-Mortalität
Shin et al. (2024; [13])	Südkorea	Retrospektive Kohortenstudie, Single-Center-Studie	3949	≥ 18 Jahre, kardiologische ITS	Ungeplante Wiederaufnahme oder Tod auf Normalstation nach ITS-Entlassung	4,7 % (*n* = 184)	Erhöhter SOFA-Score bei ITS-Entlassung; Herzinsuffizienz; Übergewicht/Adipositas	Nicht berichtet	Erhöhte Ereignisrate (Wiederaufnahme oder Tod auf Normalstation)
Son et al. (2021; [14])	Südkorea	Retrospektive Kohortenstudie, Single-Center-Studie	739	≥ 18 Jahre, internistische ITS	Wiederaufnahme während desselben Krankenhausaufenthalts	8,9 % (*n* = 66)	Prolongierte invasive Beatmung (> 14 Tage); kontinuierliches Nierenersatzverfahren	Respiratorische Dekompensation; Multiorganversagen	Erhöhte Krankenhausmortalität
Terrington et al. (2025; [15])	UK	Retrospektive Kohortenstudie, Single-Center-Studie	1393	≥ 18 Jahre, interdisziplinäre ITS	Wiederaufnahme oder Tod auf Normalstation nach ITS-Entlassung	5,3 % (*n* = 74)	Reduziertes Bewusstseinsniveau; erhöhte Herzfrequenz; erhöhter SOFA-Score bei Entlassung	Hypoxie; Sepsis; hämodynamische Instabilität; neurologische Verschlechterung	Erhöhte Ereignisrate (Wiederaufnahme oder Tod auf Normalstation)
Yang et al. (2025; [17])	China	Retrospektive Kohortenstudie, Single-Center-Studie	3028	≥ 18 Jahre, Patienten nach Ösophagusoperation, chirurgische ITS	Wiederaufnahme während desselben Krankenhausaufenthalts	3,6 % (*n* = 110)	Alter ≥ 75 Jahre; längere Operationsdauer; präoperative Hypalbuminämie; reduzierte DLCO	Respiratorische Komplikationen; Sepsis/septischer Schock; postoperative Blutung; Reoperation	Erhöhte 90-Tage-Mortalität; erhöhte Reoperationsrate

^1^Bei Amagai et al. (2025; [2]) bezieht sich die angegebene Wiederaufnahmerate auf ungeplante Wiederaufnahmen während desselben Krankenhausaufenthalts insgesamt; die Zeitintervalle ≤ 24 h, ≤ 48 h und ≤ 72 h wurden in der Originalarbeit als Subanalysen berichtet. ^2^In mehreren Studien wurde ein längerer initialer ITS-Aufenthalt als Risikofaktor identifiziert; die konkrete Verweildauer war jedoch nicht in allen Studien vergleichbar berichtet. *DLCO* pulmonale Diffusionskapazität, *ITS* Intensivstation, *n* Population, *SOFA* Sequential Organ Failure Assessment, *UK* United Kingdom

### Risikofaktoren einer Wiederaufnahme auf die Intensivstation

Die eingeschlossenen Studien zeigen, dass ungeplante Wiederaufnahmen auf die ITS nicht isoliert auftreten, sondern sich im Kontext eines Zusammenspiels patientenbezogener, krankheitsbezogener und versorgungsbezogener Einflussfaktoren erklären lassen [1–3, 13, 14, 17].

#### Patientenbezogene Faktoren

Verschiedene patientenbezogene Merkmale, die mit einer Wiederaufnahme auf die ITS assoziiert waren, konnten identifiziert werden [2, 3, 17]. Einen wesentlichen patientenbezogenen Faktor stellte ein höheres Lebensalter dar. Patienten mit Wiederaufnahme waren im Mittel älter [2]. Ein Alter ≥ 75 Jahre war mit einer erhöhten Wahrscheinlichkeit für eine Wiederaufnahme verbunden (Odds Ratio 2,12; [17]).

Neben dem chronologischen Alter wurde der funktionelle Status als relevanter Faktor identifiziert. Die Unfähigkeit zum Zeitpunkt der Intensiventlassung, selbstständig zu stehen, war adjustiert für Alter und Komorbiditäten mit einer erhöhten Wahrscheinlichkeit einer späteren Wiederaufnahme verbunden (Odds Ratio 1,85; [3]).

Darüber hinaus wurden kognitive Veränderungen als Einflussfaktoren beschrieben. Das Auftreten eines Delirs während des initialen Intensivaufenthalts war mit einer erhöhten Wiederaufnahmerate verbunden (adjustiertes relatives Risiko 2,59; 95 %-Konfidenzintervall [KI] 2,07–3,26; [1]).

#### Krankheitsbezogene Faktoren

Neben patientenbezogenen Merkmalen wurden krankheitsbezogene Faktoren identifiziert, die mit einer Wiederaufnahme auf die ITS in Zusammenhang stehen. Die krankheitsbezogenen Faktoren beziehen sich dabei hauptsächlich auf Parameter von Organfunktionsstörungen (gut quantifizierbar durch den Sequential-Organ-Failure-Assessment[SOFA]-Score), spezifische Komplikationen während des initialen Intensivaufenthalts sowie bereits bei Aufnahme bestehende klinische Merkmale wie Herzinsuffizienz oder Adipositas. Ein höherer SOFA-Score zum Zeitpunkt der Intensiventlassung als Ausdruck fortbestehender Organfunktionsstörungen ist mit einer erhöhten Wahrscheinlichkeit für eine erneute Aufnahme assoziiert (Odds Ratio 1,20 pro Punkt; [13]).

Patienten, bei denen während des initialen Intensivaufenthalts eine Aspiration auftrat, hatten ein deutlich erhöhtes Risiko für eine Wiederaufnahme auf die ITS (relatives Risiko 3,0). Hinsichtlich der Art der Aspiration, beispielsweise in Bezug auf Mikroaspiration oder Aspiration von Mageninhalt, wurde dabei nicht differenziert [1].

In chirurgischen Populationen scheint der präoperative Gesundheitszustand ebenfalls einen Einfluss auf die Wiederaufnahmeraten zu haben. Präoperative Hypalbuminämie sowie eine eingeschränkte pulmonale Diffusionskapazität scheinen hier eine Rolle zu spielen [17].

#### Versorgungsbezogene Faktoren

Neben patienten- und krankheitsbezogenen Merkmalen wurden versorgungsbezogene Faktoren identifiziert. In mehreren Studien zeigten multivariate Analysen eine längere Dauer des initialen Intensivaufenthalts bei Patienten mit späterer Wiederaufnahme, was sowohl in interdisziplinären als auch in spezialisierten intensivmedizinischen Settings beobachtet wurde. Eine differenzierte Analyse eines möglichen nichtlinearen Zusammenhangs zwischen sehr kurzer Verweildauer und Wiederaufnahmerisiko wurde in den eingeschlossenen Arbeiten nicht berichtet [2, 3].

Art und Dauer organunterstützender Maßnahmen waren ebenfalls mit der Wiederaufnahme assoziiert. Eine prolongierte invasive Beatmung von mehr als 14 Tagen war mit einer erhöhten Wiederaufnahmerate verbunden (Odds Ratio 4,77–13,25), ebenso der Einsatz kontinuierlicher Nierenersatzverfahren (Odds Ratio 4,57; [14]).

Auch perioperative Versorgungsaspekte konnten identifiziert werden. So war eine längere Operationsdauer mit einer erhöhten Wiederaufnahmerate verbunden (Odds Ratio 1,15 pro Stunde; [17]).

### Ursachen der Wiederaufnahme auf die Intensivstation

Die eingeschlossenen Studien berichteten neben assoziierten Risikofaktoren auch über die klinischen Ursachen bzw. Indikationen, die zur Wiederaufnahme auf die ITS führten [1, 2, 10, 11, 14, 15, 17]. Erfassung und Darstellung der Ursachen der Wiederaufnahme waren zwischen den Studien uneinheitlich. Während einige Arbeiten detaillierte Angaben zu den zugrunde liegenden Indikationen machten, wurden in anderen Studien keine differenzierten Angaben zu Ursachen berichtet [3, 13]. Zudem erfolgte nicht in allen Studien eine konsistente prozentuale Aufschlüsselung der zugrunde liegenden Diagnosen.

#### Respiratorische Ursachen

Respiratorische Komplikationen stellten in mehreren Studien die häufigste Ursache einer Wiederaufnahme dar. Dies konnte unabhängig vom intensivmedizinischen Setting festgestellt werden [1, 10, 11, 14, 15, 17]. Die berichtete Inzidenz respiratorischer Ursachen lag in den untersuchten Kohorten zwischen 33 % und 66,4 % der Wiederaufnahmen [11, 17]. Die respiratorischen Komplikationen wurden dabei nicht weiter differenziert, sondern allgemein als respiratorische Verschlechterung bzw. Dekompensation oder Hypoxie beschrieben [1, 10, 11, 14, 15, 17]. Aspirationsereignisse wurden ebenfalls als respiratorische Ursachen eingeordnet [1].

#### Kardiovaskuläre und hämodynamische Ursachen

Kardiovaskuläre und hämodynamische Instabilitäten wurden ebenfalls als relevante Ursachen der Wiederaufnahme identifiziert [2, 10]. In der multizentrischen Analyse von Amagai et al. (2025; [2]) benötigten 26,1 % der wiederaufgenommenen Patienten eine hämodynamische Unterstützung mit Vasopressoren, verglichen mit 23,1 % während des initialen Intensivaufenthalts, was eine klinisch relevante hämodynamische Verschlechterung bei Wiederaufnahme widerspiegelt. In der Studie von Moore et al. (2025; [10]) wurden kardiovaskuläre Instabilitäten als häufige Indikation für eine erneute intensivmedizinische Betreuung beschrieben, jedoch ohne weitere quantitative oder ätiologische Differenzierung.

#### Infektiöse Ursachen

Infektiöse Komplikationen, einschließlich Sepsis und septischem Schock, wurden insbesondere in chirurgischen und interdisziplinären Kohorten als Ursachen der Wiederaufnahme beschrieben [15, 17]. In der Studie von Yang et al. (2025; [17]) entfielen 13,6 % der Wiederaufnahmen auf septische Krankheitsbilder nach Ösophagusresektionen.

#### Neurologische Ursachen

Neurologische Verschlechterungen stellten in einzelnen Studien eine weitere Ursache der Wiederaufnahme dar [10, 15]. Diese wurden überwiegend als unspezifische neurologische Verschlechterungen bzw. Bewusstseinsveränderungen beschrieben, ohne weiterführende Differenzierung [10, 15].

#### Weitere Ursachen

Weitere, seltener berichtete Ursachen waren postoperative Blutungen sowie prozedur- oder operationsassoziierte Komplikationen. Diese traten vor allem in chirurgischen Patientenkollektiven auf [11, 17].

### Einfluss einer Wiederaufnahme auf das Patientenoutcome

Die eingeschlossenen Studien untersuchten den Zusammenhang zwischen Wiederaufnahmen auf die ITS und klinischem Outcome. Im Fokus standen dabei insbesondere Mortalität, Länge des Krankenhaus- und Intensivaufenthalts sowie der intensivmedizinische Ressourcenbedarf [1–3, 11, 13–15, 17].

#### Krankenhaus- und Intensivstationsmortalität

Eine erhöhte Mortalität bei Patienten mit ITS-Wiederaufnahme zeigte sich konsistent über alle eingeschlossenen Studien hinweg, wobei zwischen Krankenhaus- und ITS-Mortalität differenziert wurde [1–3, 11, 13–15, 17].

In der Studie von Padkins et al. (2022; [11]) betrug die Krankenhausmortalität bei Patienten mit ITS-Wiederaufnahme 24 %, verglichen mit 3 % bei Patienten ohne erneuten ITS-Aufenthalt. Während des Aufenthalts auf der ITS nach Wiederaufnahme verstarben 7,7 % der Patienten. Darüber hinaus war die ITS-Wiederaufnahme mit einer erhöhten 1‑Jahres-Mortalität assoziiert (Hazard Ratio 1,32). Die Studie von Terrington et al. (2025; [15]) lieferte vergleichbare Ergebnisse. Bei Patienten mit dem kombinierten Endpunkt aus Wiederaufnahme auf die ITS oder Tod auf der Normalstation betrug die Krankenhausmortalität 24,3 %, verglichen mit einer Mortalität von 3,1 % bei Patienten ohne Ereignis.

In chirurgischen Populationen wurde zudem eine erhöhte 90-Tage-Mortalität nach Wiederaufnahme beschrieben [17].

#### Länge des Krankenhausaufenthalts

Eine verlängerte Krankenhausverweildauer bei Patienten mit ITS-Wiederaufnahme wurde in mehreren Studien beobachtet [10, 11, 15]. In der Studie von Padkins et al. (2022; [11]) betrug die mediane Krankenhausverweildauer 27 Tage bei Patienten mit Wiederaufnahme, verglichen mit 9 Tagen bei Patienten ohne Wiederaufnahme. Auch Moore et al. (2025; [10]) berichteten eine signifikant verlängerte Krankenhausverweildauer bei wiederaufgenommenen Patienten (Median 23 vs. 12 Tage). In der Studie von Terrington et al. (2025; [15]) zeigte sich ebenfalls eine längere Krankenhausverweildauer bei Patienten mit dem kombinierten Endpunkt aus Wiederaufnahme oder Tod auf der Normalstation im Vergleich zur ereignisfreien Kohorte.

Darüber hinaus ging der klinische Verlauf mit einem erhöhten Behandlungs- und Ressourcenbedarf einher [10, 11].

#### Länge des zweiten Intensivaufenthalts

Eine erneute Aufnahme auf die ITS war mit einer verlängerten intensivmedizinischen Behandlungsdauer assoziiert. Dies wurde insbesondere auf eine höhere Krankheitslast und den erhöhten Bedarf an organunterstützenden Maßnahmen, insbesondere mit prolongierter invasiver Beatmung, zurückgeführt [10, 11, 15].

## Diskussion

Der vorliegende Übersichtsbeitrag zeigt, dass ungeplante Wiederaufnahmen auf die ITS während desselben Krankenhausaufenthalts kontextübergreifend mit ungünstigen klinischen Outcomes verbunden sind. Die Wiederaufnahmeraten variierten zwischen 3,6 % und 9,1 % in Abhängigkeit von Patientenpopulation, intensivmedizinischem Setting und methodischer Definition der Wiederaufnahme. Wiederaufnahmen waren mit einer erhöhten Krankenhaus- und ITS-Mortalität, verlängerten Krankenhaus- und Intensivaufenthalten sowie einem erhöhten intensivmedizinischen Ressourcenverbrauch assoziiert. Diese Assoziationen zeigten sich in unterschiedlichen Patientenkollektiven, wenngleich mit unterschiedlicher Ausprägung [1–3, 10, 11, 13–15, 17].

Wiederaufnahmen auf die Intensivstation sind nicht monokausal zu erklären

Die Ergebnisse verdeutlichen, dass Wiederaufnahmen auf die ITS nicht monokausal, sondern Ausdruck eines komplexen Zusammenspiels aus patientenbezogenen, krankheitsbezogenen und versorgungsbezogenen Faktoren sind. Zu den konsistent berichteten patientenbezogenen Risikofaktoren zählten ein höheres Lebensalter, funktionelle Einschränkungen zum Zeitpunkt der Intensiventlassung sowie kognitive Störungen wie Delir [1–3, 17].

Krankheitsbezogen erwiesen sich insbesondere persistierende Organfunktionsstörungen als relevant. Ein erhöhter SOFA-Score bei Intensiventlassung stellte einen Prädiktor für eine Wiederaufnahme dar, was die prognostische Bedeutung des klinischen Zustands zum Zeitpunkt der Entlassung unterstreicht [13]. Spezifische Komplikationen wie Aspirationsereignisse waren mit einem deutlich erhöhten Wiederaufnahmerisiko verbunden, wenngleich eine differenzierte Erfassung der resultierenden Störungen häufig fehlte [1].

Diese Risikofaktorenperspektive ergänzt die Analyse klinischer Ursachen der Wiederaufnahme. Respiratorische Komplikationen stellten in nahezu allen untersuchten Settings die häufigste Indikation für eine erneute intensivmedizinische Aufnahme dar, gefolgt von hämodynamischer Instabilität, infektiösen Komplikationen und neurologischen Verschlechterungen [1, 10, 11, 15, 17]. Diese Befunde sprechen dafür, dass Wiederaufnahmen häufig mit einer klinischen Dekompensation zentraler Organsysteme assoziiert sind, und verschiebt die Perspektive von einer reinen Ursachenbeschreibung hin zur Frage einer Risikostratifizierung und Entscheidungsfindung im Rahmen der ITS-Entlassung. Versorgungsbezogene Faktoren, wie eine längere Dauer des initialen Intensivaufenthalts, prolongierte invasive Beatmung oder der Einsatz kontinuierlicher Nierenersatzverfahren, waren ebenfalls mit einer erhöhten Wiederaufnahmerate verbunden [2, 3, 14]. Diese Parameter reflektieren nicht primär eigenständige Ursachen, sondern vielmehr die zugrunde liegende Krankheitsschwere und die Komplexität des Behandlungsverlaufs.

Die aktuellen Daten bestätigen die Assoziation zwischen Wiederaufnahme und erhöhter Mortalität

Die Ergebnisse des vorliegenden Übersichtsbeitrags stehen im Einklang mit früheren Arbeiten, welche die Wiederaufnahmen auf die ITS als Marker einer erhöhten Vulnerabilität und eines ungünstigen klinischen Verlaufs beschrieben haben. Bereits frühere Studien identifizierten eine erhöhte Krankheitsschwere bei Intensiventlassung sowie persistierende Organfunktionsstörungen als zentrale Prädiktoren für eine Wiederaufnahme [4, 5, 7, 8]. Auch die Assoziation zwischen höherem Lebensalter und Wiederaufnahmerisiko wurde bereits in früheren Arbeiten beschrieben [5, 16]. Die im Manuskript dargestellten Ergebnisse erweitern diese Erkenntnisse, indem sie zusätzlich funktionelle und kognitive Aspekte berücksichtigen. Insbesondere der funktionelle Status bei Intensiventlassung zeigt sich als unabhängiger Risikofaktor für eine Wiederaufnahme [3].

Hinsichtlich des Outcomes bestätigen die aktuellen Daten die wiederholt beschriebene Assoziation zwischen Wiederaufnahme und erhöhter Mortalität [5, 9]. Gleichzeitig zeigen populationsspezifische Unterschiede, dass die prognostische Bedeutung einer Wiederaufnahme je nach Patientenkollektiv variiert. Während insbesondere internistische und kardiologische Intensivpopulationen eine deutlich erhöhte Kurz- und Langzeitmortalität aufweisen [11, 15], sind die Ergebnisse in traumachirurgischen Populationen heterogener [1, 17].

Zusammenfassend bestätigen die vorliegenden Ergebnisse zentrale Annahmen der bisherigen Literatur, erweitern diese jedoch durch die Berücksichtigung funktioneller, kognitiver und versorgungsbezogener Faktoren. Die Daten sprechen dafür, Wiederaufnahmen auf die ITS nicht als isoliertes Qualitätsmerkmal zu betrachten, sondern als Ausdruck eines komplexen Zusammenspiels aus Patientenvulnerabilität, Krankheitsdynamik und intensivmedizinischen Versorgungsentscheidungen.

## Limitationen

Der vorliegende Übersichtsbeitrag unterliegt mehreren Limitationen. Die Analyse basiert ausschließlich auf retrospektiven Kohortenstudien, wodurch kausale Schlussfolgerungen nicht möglich sind. Die identifizierten Assoziationen zwischen Wiederaufnahme, Risikofaktoren und Outcome spiegeln daher Zusammenhänge wider, ohne kausale Rückschlüsse zuzulassen.

Es zeigte sich eine ausgeprägte methodische Heterogenität zwischen den eingeschlossenen Studien. Unterschiede bestanden insbesondere hinsichtlich

- der Definition von Wiederaufnahmen,
- der zeitlichen Abgrenzung (beispielsweise ≤ 24 h, ≤ 48 h, ≤ 72 h) und
- der Erfassung von Outcomeparametern.

Diese Heterogenität limitiert die direkte Vergleichbarkeit der berichteten Wiederaufnahmeraten und Outcomes.

Die Literaturrecherche erfolgte in zwei Datenbanken (PubMed und CINAHL), wobei das Screening von einem einzelnen Reviewer durchgeführt wurde. Obwohl ein strukturierter Such- und Auswahlprozess angewendet wurde, kann ein Selektions- oder Publikationsbias nicht vollständig ausgeschlossen werden.

Schließlich war eine quantitative Synthese der Ergebnisse aufgrund der beschriebenen Heterogenität nicht möglich, sodass die Ergebnisse ausschließlich narrativ zusammengefasst wurden. Dies limitiert die Möglichkeit, Effektstärken über Studien hinweg systematisch zu vergleichen.

## Fazit für die Praxis

- Ungeplante Wiederaufnahmen auf die Intensivstation sind ein relevantes Ereignis in unterschiedlichen Versorgungssettings.
- Wiederaufnahmen sind klinisch hochrelevant, da sie mit einer erhöhten Mortalität sowie mit verlängerten Krankenhaus- und Intensivaufenthalten assoziiert sind.
- Wiederaufnahmen binden zusätzliche intensivmedizinische Ressourcen und gehen mit einem erhöhten Bedarf an Organunterstützung einher.
- Das Wiederaufnahmerisiko spiegelt ein Zusammenspiel aus Patientenvulnerabilität, persistierender Krankheitsschwere und Versorgungsfaktoren wider.
- Eine strukturierte Gestaltung der Intensiventlassung und der postintensivmedizinischen Betreuung sollte insbesondere bei Patienten mit erhöhtem Wiederaufnahmerisiko berücksichtigt werden.
